# The impact of advanced paternal age on the live birth rate in patients undergoing Assisted Reproduction treatment: Findings from an analysis at a public reproductive center in Brazil

**DOI:** 10.5935/1518-0557.20240053

**Published:** 2024

**Authors:** Vanessa B.M. Maia, Aline Q. Rodrigues, Victor E.T. Sousa, Mariana F.R. Barcelos, Jair T. Goulart, Fernanda Paulini, Natalia I.Z. Tierno

**Affiliations:** 1 Maternal and Child Hospital of Brasilia Dr. Antônio Lisboa, Assisted Reproduction Sector, Brasilia-DF, 70203-900, Brazil; 2 University of Brasilia, Institute of Biological Sciences, Department of Physiological Sciences, Brasilia-DF, 70910-900, Brazil

**Keywords:** age fertility, assisted reproductive technology, full-term births, reproductive parameters, infertility

## Abstract

**Objective:**

Human reproduction presents a challenge for our species, as evidenced by the escalating rates of infertility. This trend has prompted inquiries into diverse strategies aimed at mitigating infertility and enhancing conception rates. Despite the extensive research on advanced maternal age as a risk factor for reproductive outcomes, paternal age has historically garnered comparatively less attention. The aim of this study was to assess the impact of paternal age on embryos and its subsequent repercussions on fertilization rate, biochemical pregnancy, clinical pregnancy, and live birth rate in individuals undergoing assisted reproductive treatment in a public reproductive center located in Brazil.

**Methods:**

This investigation adopted a retrospective cohort, cross-sectional, analytical design, utilizing the analysis of secondary data, covering the period from July 2015 to July 2021.

**Results:**

A total of 350 couples grappling with infertility and undergoing intrauterine insemination (IUI), *in vitro* fertilization (IVF), and intracytoplasmic sperm injection (ICSI) were included in the analysis. Examination of age groups revealed a notable correlation between the ages of women and men (correlation coefficient R=0.12, p<0.0001). In the analysis of IVF techniques, a discernible trend towards a negative correlation with paternal age was observed, signifying that higher paternal age was linked to lower fertilization rates (p=0.004).

**Conclusions:**

Advanced paternal age significantly impacts full-term birth rates in IVF procedures, emphasizing the need for preconception public health advisories that underscore the risks associated with delaying parenthood for both men and women, particularly among those necessitating assisted reproductive techniques.

## INTRODUCTION

Human reproduction, in its natural conception, constitutes a challenging process for the human species. As documented in the literature, approximately 80% of couples will achieve conception within the initial 6 months of attempting pregnancy, with monthly fecundability (the probability of pregnancy per month) being notably higher during the initial three months (Gnoth *et al.*, 2003). Nevertheless, infertility rates exhibit an ascending trajectory.

Infertility is defined as the incapacity to conceive following a period of 12 months of regular unprotected sexual intercourse. This condition affects approximately 10 to 15% of couples throughout their reproductive trajectories (Lu *et al.*, 2023). One approach to mitigate infertility and enhance conception rates involves the utilization of assisted reproductive techniques, encompassing highly intricate treatments such as *in vitro* fertilization (IVF) (Jain & Singh, 2024).

The standard initial assessment of infertility, in line with established guidelines, involves confirming normal ovulatory function, a semen analysis within normal parameters, and at least one intact fallopian tube. However, it is crucial in clinical practice to recognize the inherent limitations of this diagnostic evaluation, as it is susceptible to both overdiagnosis and underdiagnosis of the underlying causes of infertility (Buckett & Sierra, 2019; Lu *et al.*, 2023).

Infertility is increasingly associated with advanced maternal age, a well-explored risk factor for reproductive outcomes (du Fossé *et al.*, 2020). The reproductive risks linked to advanced maternal age, typically defined as 35 years or older, are an essential aspect of preconception counseling and are widely recognized by the general population (Heffner, 2004). In contrast, paternal age has been historically overlooked in studies assessing the age-related impact on reproductive outcomes. Nevertheless, its potential role has recently gained attention and is now the subject of intensive investigation.

Recent studies have revealed a consistent increase in average paternal age by 3.5 years, observed across all races, ethnicities, and regions, regardless of educational levels (Khandwala *et al.*, 2017). Multiple factors contribute to this trend, including delayed marriage, extended life expectancy, the accumulation of financial capital, a growing inclination to postpone family formation for postgraduate and higher education pursuits, and the establishment of a suitable career. Consequently, these elements collectively contribute to the overall rise in paternal age (Halvaei *et al.*, 2020).

In fact, the biological clock of men, given their continuous sperm production throughout their lives, has been undervalued compared to that of women (Polastri *et al.*, 2021). Establishing a clear correlation between paternal age and adverse outcomes in assisted reproductive technologies (ART) is challenging due to the complexity of isolating the father’s age from maternal factors and other confounding variables (Farabet *et al.*, 2023). While some studies suggest a positive association between advanced paternal age and unfavorable ART outcomes, no well-defined age threshold has been validated in these studies (Polastri *et al.*, 2021). Instead, contemporary insights are grounded in studies exploring alterations related to spermatogenesis and fertility.

Among studies using specific age thresholds, an age exceeding 40 years frequently stands out as a commonly selected delineation point (Ramasamy *et al.*, 2015; Polastri *et al.*, 2021). Certain authors have reported decreased pregnancy rates and live birth rates among men over 46 years (Marsidi *et al.*, 2021) or over 50 years (da Silva Bitecourt *et al.*, 2018) of age, even after accounting for maternal age and other confounding factors, in contrast to younger men. However, when the analysis was restricted to women under the age of 35, no significant difference was observed between the two male cohorts (Marsidi *et al.*, 2021).

In 2018, Oldereid *et al.* (2018) conducted a comprehensive assessment of paternal factors’ influence on various perinatal and pediatric outcomes. The results revealed associations between advanced paternal age and adverse consequences in offspring, particularly emphasizing psychiatric disorders, stillbirths, and various congenital defects. The substantial relationship between paternal age and the mutation rate in offspring may be attributed to the increased number of germline divisions observed in older males (Kong *et al.*, 2012). Alongside an increased prevalence of point mutations, evidence supports the association between advanced paternal age and phenomena such as DNA strand breaks in spermatozoa, errors in genetic imprinting, and chromosomal anomalies, all contributing to episodes of spontaneous abortion (Sartorius & Nieschlag, 2010; Robinson *et al.*, 2012; Kobayashi *et al.*, 2017).

As outlined by Halvaei *et al.* (2020), advanced paternal age is linked to significant reductions in various sperm characteristics, including seminal volume, sperm count, motility, morphology, and viability. The direct cause of this relationship between these variables and male age remains unknown, but several potential mechanisms undergo alterations with advancing age. These include diminished functionality of the reproductive accessory glands, cellular and physiological changes such as reduced capacity for cellular and tissue damage repair, decreased germ cell count and androgen levels, as well as structural changes in male reproductive anatomy, including narrowing of seminiferous tubules, vascular insufficiency, and systemic conditions associated with aging (Eskenazi *et al.*, 2003; Belloc *et al.*, 2014; Gunes *et al.*, 2016).

Acknowledging that the father contributes 50% of the embryonic genetic material, exploring paternal age can enhance understanding of the complex process of gestational loss and its effect on the live birth rate for individuals undergoing assisted reproductive treatment. Consequently, this study aimed to evaluate the influence of paternal age on embryos, subsequently affecting fertilization rate, biochemical pregnancy, clinical pregnancy, and the live birth rate in patients undergoing assisted reproductive treatment at a public reproductive center.

## MATERIAL AND METHODS

### Ethics and patients

A retrospective cohort study was conducted in the Assisted Reproduction service of the Hospital Materno Infantil de Brasília (HMIB) Dr. Antônio Lisboa, a public health institution located in Brasilia, Brazil, according to consubstantiation opinion nº 5.733.378/CAAE: 61859922.00000.5553. A comprehensive query of the database was executed to assess information from all couples undergoing assisted reproductive procedures, including *in vitro* fertilization (IVF), intracytoplasmic sperm injection (ICSI), fresh embryo transfer (ET), and intrauterine insemination (IUI) during the period from July 2015 to July 2021.

The research participants consisted of couples diagnosed with female or male infertility or both. The selected population adhered to specific criteria for women, namely age under 40 years and a body mass index (BMI) between 18 and 29. The corresponding male partners of these women were also included. The data were collected and exported to a Microsoft Excel spreadsheet, version 2016. The primary outcome-related data were meticulously recorded, taking into consideration the maternal and paternal age at the time of ovarian stimulation, the number of antral follicles at the beginning of the study, and semen analysis parameters, including quantity, morphology, and motility. Additionally, the number of retrieved oocytes, the number of transferred embryos, and the clinical outcomes of pregnancy were documented. These outcomes encompassed the fertilization rate, the occurrence of biochemical pregnancy, clinical pregnancy, and the rate of live births.

Patients with a history of severe infertility due to male factors (sperm concentration <5 million/ml), use of donor semen, utilization of frozen sperm, or the need for epididymal or testicular biopsy for sperm retrieval were excluded from the study. Additionally, cycles involving donor oocytes were excluded to facilitate the analysis of the impact of male age on subfertile women of varying ages using autologous oocytes. Cycles that led to embryo storage without subsequent transfer were also excluded from the analysis.

### Hormonal parameters and protocols

In the pre-IVF assessment of patients, a comprehensive series of examinations was conducted. For female participants, these included evaluations such as TSH and prolactin dosage, blood typing, and Rh factor determination, along with serological tests for cytomegalovirus, toxoplasmosis, rubella, chagas disease, hepatitis B and C, HIV I and II, Human T-cell lymphotropic virus (HTLV) I and II, Venereal Disease Research Laboratory (VDRL), antral follicle count, and oncotic colpocytopathology. In the case of male subjects, in addition to the analyses, a spermogram and sperm culture with antibiogram were performed.

Ovarian stimulation protocols were implemented in accordance with specific medical criteria, with medication dosage tailored to align with the patient’s age and antral follicle count (AFC):

- Patients up to 29 years: 150 to 225IU
- From 30 to 35 years: 150 to 300IU
- Above 36 years: 150 to 300IU or as per medical guidance
- Patients with Polycystic Ovary Syndrome (PCOS): initiate with 150IU or as per medical guidance

Following the baseline ultrasound, if the ovaries are quiescent (preferably with the largest follicle ≤9mm), daily administration of stimulation medication commenced until the follicles reached the desired diameter. Stimulation medications included:

- Menotropin: Menopur (Ferring GmbH, Germany) and Merional (IBSA Institut Biochimique, Swiss)
- Urofollitropin (highly purified FSH): Fostimon (IBSA Institut Biochimique, Swiss)
- FSH-r: Gonal (alfa-folitropin- Merck, Italy)

When the follicles reached 16 - 22 mm in diameter, the TRIGGER was performed using hCG (Ovidrel 6500IU/mL- Merck, Italy) or Choriomon 5000IU or 10.000IU (IBSA Institut Biochimique, Swiss) or duo TRIGGER hCG + GnRH agonist (Gonapeptyl daily- Ferring GmbH, Germany). After 35 hours of TRIGGER, the ovarian follicle retrieval and semen collection were carried out.

For oocyte collection, patients presented to the Human Reproduction Laboratory in a fasting state. Oocyte retrievals were conducted by the clinic’s medical staff using 17-gauge single-lumen needles (CooperSurgical^®^ Wallace^®^ Single Lumen Oocyte Recovery System, USA) under the guidance of endovaginal ultrasound (Logic GE^®^ P5). Prior to collection, the needles were washed with heparinized culture medium (phosphate-buffered saline- Ingamed, Maringá, Brazil) supplemented with 25 UI/ml of heparin (Cristalia- São Paulo, Brazil) at a temperature range between 25-37°C, with a pH maintained around 7.3±0.1 and an osmolarity range between 280-288 mOsm/L. The follicular fluid was aspirated using 20mL luer-lock syringes attached to the needle. As the syringes became filled, they were replaced with empty ones, and the collected follicular fluid was promptly transported to the laboratory. There, it underwent analysis through stereo microscope observation to detect the presence of the cumulus oophorus complex.

Semen examinations, analyses, and processing adhered to the sperm analysis protocols and parameters established by the World Health Organization (WHO) in 2021 (WHO, 2021). Semen samples were subjected to liquefaction on a warm plate within a Class 2 safety cabinet (Heraeus HeraSafe HS18, Germany) for approximately 20 minutes before analysis. The comprehensive analysis encompassed parameters such as abstinence, volume, concentration, motility, and morphology. For sample preparation, the Irvine Scientific^®^ ISolate^®^ density gradient kit (FUJIFILM Irvine Scientific, USA) was employed, followed by centrifugation. Subsequently, the sperm pellets were resuspended in pre-equilibrated fertilization medium (FM), undergoing a second and third round of centrifugations tailored to the sperm count. The prepared samples were incubated at 37°C with 6% CO_2_ until the time of insemination. For *in vitro* fertilization, oocytes were incubated at 37°C with 6% CO_2,_ occurring in an *in vitro* fertilization chamber approximately 40-42 hours after hCG triggering. The sperm suspension for insemination was calculated at approximately 70,000 motile sperm per well.

A biochemical pregnancy was identified by a positive human chorionic gonadotropin (Beta-hCG) test, with levels surpassing the laboratory’s predefined benchmark (values >0.2mIU/mL, performed 14 days post Embryo Transfer or Intrauterine Insemination). Clinical pregnancy was ascertained through the visualization of an amniotic sac on ultrasound, while the live birth rate was calculated as the occurrence of a live birth following a pregnancy lasting a minimum of 37 weeks’ gestation. In this context, a live birth signifies the complete expulsion or extraction of the fertilized fetus from the woman after 22 completed weeks of gestation, manifesting signs of life such as respiratory movements, heartbeat, umbilical cord pulsation, or distinct voluntary muscle movements, irrespective of whether the umbilical cord is severed or the placenta remains attached. It’s crucial to note that live births are individualized, so a twin birth is considered equivalent to two live births. Conversely, a miscarriage is defined as the spontaneous termination of a pregnancy before reaching 22 completed weeks of gestation.

The study results are based on a single *in vitro* fertilization/intracytoplasmic sperm injection (IVF/ICSI) cycle per patient with embryo transfer (ET). The number of embryos transferred per attempt varied from one to three embryos. It’s important to note that the ploidy status of the embryos remained unknown, as no genetic studies were conducted.

### Statistical analyses

We performed multiple regression analysis using GraphPad Prism 9.0 software (GraphPad, San Diego, USA) to assess the relationship while controlling for maternal age. Given that maternal age is correlated with paternal age and has a known impact on the outcomes evaluated in our study, we addressed this confounding factor by incorporating maternal age as a covariate in our statistical models. Specifically, for the parameter “fertilization rate,” representing a continuous variable ranging from 0 to 1, we employed multiple linear regression. Similarly, for the binary categorical variables “Beta-hCG,” “presence of gestational sacs,” and “full-term birth,” denoting the presence or absence of specific conditions, we utilized multiple logistic regression. In all cases, the effect of the variable on the outcome was considered statistically significant when *p*<0.05.

## RESULTS

Throughout the study period, a total of 1291 ART procedures were conducted. These included 212 Transcervical Embryo Transfer (TEC) procedures, 209 Intrauterine Insemination (IUI) procedures, 65 *In Vitro* Fertilization (IVF) procedures, and 92 Intracytoplasmic Sperm Injection (ICSI) procedures. Following careful assessment, only IVF, ICSI, and IUI procedures were considered suitable for analysis, ensuring that all included samples were performed within the designated time frame. Subsequently, after applying exclusion criteria, a cohort of 350 patients was included in the statistical analysis.

Among the couples participating in the study, 8.5% exclusively presented male factor infertility, while 47.4% solely exhibited female factor infertility. Additionally, 44% of couples manifested a combination of both factors. Regarding female etiologies of infertility, 23.7% of patients were attributed to tubal factors, 15.5% to ovulatory factors, 5.7% to endometriosis, 5.1% to uterine factors, and 4.5% to other causes.

When assessing age groups, a robust correlation was identified between the ages of women and men, as indicated by a correlation coefficient (R) of 0.12, with statistical significance (*p*<0.0001). Table 1 outlines the maternal and paternal age ranges of the patients included in the study, while Table 2 presents the statistical outcomes for each procedure.

**Table 1:** Analysis of maternal and paternal age distribution in the study’s target population across assisted reproductive technology (ART) procedures: minimum and maximum age, mean and standard deviation.

Intrauterine insemination (IUI)												
	**Beta-hCG [positive(≤0.2mUI/ml) / negative (<0.2mUI/ml)]**			**Gestacional sacs** **(presence/ absence)**						**Full-term births** **(yes/no)**		
	**|Z| value**	***p* value**	**Significance**	**|Z| value**		***p* value**		**Significance**		**|Z| value**	***p* value**	**Significance**
Age (years) Maternal Paternal	1045.0 0.44	0.29 0.65	ns ns	1022.0 0.69		0.30 0.48		ns ns		1983.0 1281.0	0.04 0.2	^*^ ns
**In vitro fertilization (IVF)**												
	**Beta-hCG [positive(≤0.2mUI/ml) / negative (<0.2mUI/ml)]**			**Gestacional sacs** **(presence/ absence)**			**Full-term births (yes/no)**			**Fertilization rate** **(ranging from 0-1)**		
	**|Z| value**	***p* value**	**Significance**	**|Z| value**	***p* value**	**Significance**	**|Z| value**	**p value**	**Significance**	**|Z| value**	***p* value**	**Significance**
Age (years) Maternal Paternal	0.41 0.84	0.67 0.39	ns ns	1263.0 0.61	0.20 0.53	ns ns	1517.0 2032.0	0.12 0.04	ns ^*^	0.33 1288.0	0.73 0.20	ns ns
Intracytoplasmic sperm injection (ICSI)												
	**Beta-hCG [positive(≤0.2mUI/ml) / negative (<0.2mUI/ml)]**			**Gestacional sacs** **(presence/ absence)**			**Full-term births (yes/no)**			**Fertilization rate** **(ranging from 0-1)**		
	**|Z| value**	***p* value**	**Significance**	**|Z| value**	***p* value**	**Significance**	**|Z| value**	***p* value**	**Significance**	**|Z| value**	***p* value**	**Significance**
Age (years) Maternal Paternal	1955.0 0.55	0.05 0.57	ns ns	1603.0 0.21	0.10 0.83	ns ns	1506.0 0.00	0.13 0.99	ns ns	0.29 0.36	0.76 0.71	ns ns
IUI + IVF + ICSI												
	**Beta-hCG [positive (≤0.2mUI/ml) / negative (<0.2mUI/ml)]**			**Gestacional sacs** **(presence/ absence)**			**Full-term births (yes/no)**			**Fertilization rate** **(ranging from 0-1)**		
	**|Z| value**	***p* value**	**Significance**	**|Z| value**	***p* value**	**Significance**	**|Z| value**	***p* value**	**Significance**	**|Z| value**	***p* value**	**Significance**
Age (years) Maternal Paternal	1281.0 1527.0	0.20 0.12	ns ns	0.47 0.59	0.63 0.54	ns ns	0.00 1544.0	0.98 0.12	ns ns	0.29 1756.0	0.76 0.08	ns ns

* =significant difference (*p*<0.05) and ns=non-significant difference.

**Table 2:** Maternal and paternal age statistics across assisted reproductive technology (ART) procedures. Beta-hCG means biochemical pregnancy, gestational sac signifies clinical pregnancy and full-term births means live birth rate.

	IUI		IVF		ICSI		IUI + IVF + ICSI	
	Minimum - maximum	Mean±SD	Minimum - maximum	Mean±SD	Minimum - maximum	Mean±SD	Minimum - maximum	Mean±SD
Age (years) Maternal Paternal	25-39 23-56	34.0±3.6 37.4±6.0	26-39 25-50	34.9±3.3 35.9±4.9	26-39 26-62	35.3±2.9 38.5±5.8	25-39 23-62	34.4±3.5 37.5±5.9
**Total cases**	209		65		92		350	

IUI=Intrauterine insemination; IVF=*In vitro* fertilization; ICSI=Intracytoplasmic sperm injection. SD=Standard deviation.

In Table 2, the analysis reveals no discernible correlation between paternal and maternal ages in relation to biochemical pregnancy and clinical pregnancy within the conducted IUI procedures. Remarkably, a noteworthy trend emerges concerning the occurrence of full-term births: as maternal age increases, there is a significant decrease in the birth rate. However, it’s important to note that statistical significance was not observed for paternal age in this context.

In the analysis of IVF techniques, maternal age did not show a discernible association with the fertilization rate. However, there was a tendency towards a negative correlation with paternal age, suggesting that higher paternal age was associated with lower fertilization rates. In ICSI procedures, no significant associations were observed between the analyzed reproductive parameters and maternal or paternal ages (see Table 2).

In the comprehensive analysis of combined treatments (IUI + ICSI + IVF), no significant associations were identified with any of the parameters evaluated. This includes biochemical and clinical pregnancy rates, as well as full-term birth rates, in relation to either maternal or paternal ages.

## DISCUSSION

Infertility is recognized as a global public health issue by the World Health Organization, affecting approximately 1 in 6 individuals worldwide, irrespective of geographic or socioeconomic factors (WHO, 2023). Prevalence estimates show similarities across countries with varying income levels, standing at 17.8% for high-income countries and 16.5% for low and middle-income countries (WHO, 2023).

In Brazil, the Brazilian Society of Assisted Reproduction suggests that around 8 million individuals may experience infertility, despite legislation ensuring reproductive rights for couples. However, access to public services offering assisted reproductive technologies is limited. The 14th Report of the National Embryo Production System (SisEmbrio, 2022) indicates the presence of 172 Assisted Human Reproduction Centers in the country, with only 10 public hospitals providing these services under the Unified Health System (SUS) (Brazil, Ministry of Health, 2005; SisEmbrio, 2022).

Infertility can result from various factors in both the male and female reproductive systems, and in some cases, the causes remain unexplained. Data from the American Society for Reproductive Medicine indicate that around 20% of infertility cases are attributed solely to a male factor, while approximately 30% involve a combination of male and female factors (Practice Committee of the American Society for Reproductive Medicine, 2015).

In this study, among the included couples, 8.5% attributed infertility solely to the male factor, whereas 47.4% identified the female factor as the exclusive cause. Notably, 44% of couples recognized both male and female factors contributing to infertility. The higher prevalence of female causes observed in this population might be attributed to women seeking healthcare services more frequently, leading to more frequent diagnoses. Regarding specific causes of female infertility, the most prevalent factor in the study group was tubal problems, accounting for 23.7%, aligning with one of the most common causes reported in American data at 35% (Forman, 2018).

The findings of our study reveal a significant correlation between parental ages, indicating a strong relationship between the ages of women and men in the study. Analyzing age groups, such as pairing younger maternal age with older paternal age, has been challenging to minimize the impact of maternal age on the results. The complexity arises from the interconnected nature of advanced paternal age with advanced maternal age, given their close association. In this context, Polastri *et al.* (2021) propose that assessing the impact of male age is best accomplished by examining pregnancy outcomes in couples utilizing oocyte donation, allowing male age to be the dependent variable.

The statistical analysis uncovered a negative association between paternal age and the incidence of full-term births among patients undergoing IVF. In contrast, maternal age did not show a significant association with full-term births during IVF procedures. Consequently, from this analysis where only paternal age emerged as significant, it can be inferred that advancing paternal age adversely affects full-term birth rates, aligning with recent retrospective studies (Brandt *et al.*, 2019; Dviri *et al.*, 2020).

Studies exploring the impact of advanced paternal age on various reproductive and neonatal parameters present conflicting findings. For instance, Frattarelli *et al.* (2008) reported no association between male age and live birth rate, implantation rate, pregnancy rate or early embryo development during the cleavage stage. However, paternal age exceeding 50 years was linked to a decreased number of live births. Conversely, Alio *et al.* (2012) observed a 24% increased risk of stillbirths among babies born to parents aged 40 to 45 years compared to those born to younger parents (25-29 years). Khandwala *et al.* (2017) emphasized that parents aged 45 or older had a 14% higher likelihood of premature birth, regardless of gestational age. In a review by Brandt *et al.* (2019), additional risks associated with advanced paternal age included infertility, miscarriage, birth defects, and poor neurodevelopmental outcomes. Recent retrospective studies have indicated that advanced paternal age correlated with diminished embryo quality, reduced fertilization and pregnancy rates, and consequently, lower pregnancy and live birth rates (Dviri *et al.*, 2020).

In a retrospective cohort study involving 77,209 IVF cycles, paternal age ≥ 46 years was associated with a decreased probability of pregnancy per cycle and per transfer, as well as a lower probability of live birth. The mean maternal age in this study was 35.5±4.6 years, and the negative impact of paternal age was more pronounced among women aged ≥ 35 years. This may be attributed to maternal age serving as a stronger predictor of assisted reproductive technique outcomes (Marsidi *et al.*, 2021).

Most of these studies are retrospective, with limited representation of older male populations, potentially impacting pregnancy outcomes by excluding women of advanced maternal age. Nevertheless, a consistent finding across these studies is that the influence of paternal aging on adverse reproductive outcomes is significant but modest.

Indeed, some biological mechanisms contributing to the decline in live birth rates associated with advanced paternal age likely involve oxidative damage to sperm DNA and alterations in sperm epigenetic marks, such as methylation (Jenkins *et al.*, 2014). The integrity of human sperm DNA is crucial for both successful fertilization and normal embryonic development. Research conducted by Simon *et al.* (2014; 2019) suggests that sperm with compromised DNA integrity are inversely associated with successful pregnancies and are linked to heightened rates of miscarriage.

When evaluating the impact of paternal age on ART outcomes, it’s crucial to consider several confounding factors. Firstly, male infertility, regardless of age, significantly influences ART outcomes, highlighting the importance of assessing male factor infertility severity (Polastri *et al.*, 2021). Secondly, environmental factors such as alcohol consumption, smoking, exposure to medications with gonadotoxic effects, obesity, and other comorbidities should be considered due to their potential impact on ART outcomes (Farabet *et al.*, 2023). Thirdly, maternal age plays a significant role in determining ART outcomes and should be recognized as a confounding factor. An ideal approach to independently assess the influence of paternal age involves utilizing oocyte donors, which helps circumvent biases related to oocyte quality (Polastri *et al.*, 2021; Farabet *et al.*, 2023). Oocyte donors, typically young women without fertility issues, provide a clearer perspective. However, only a limited number of studies have investigated the effects of paternal age within the oocyte donor population, indicating a gap in research in this area.

The decision to intentionally exclude women under 40 years from our study was based on the well-documented decline in pregnancy and live birth rates after the age of 27, observed in both natural conception and assisted reproduction settings (Crawford & Steiner, 2015). Additionally, the increased incidence of aneuploidies in oocytes from women of advanced age further supported this exclusion (Bartmann *et al.*, 2004). However, despite these precautions, our analysis did not reveal any significant correlation between the variables under scrutiny and maternal age. This observation may stem from the unique characteristics of our study population, where the underlying causes of infertility among these women likely exerted a more pronounced influence on their fertility outcomes compared to age alone.

In this context, a retrospective observational study conducted by Yan *et al.* (2012) involving 11,830 IVF cycles with embryo transfer analyzed four distinct age groups: 20 to 30 years, 31 to 35 years, 36 to 40 years, and over 40 years. The findings revealed that the rates of biochemical pregnancy and clinical pregnancy were significantly lower in the older maternal age groups compared to the younger groups. Additionally, the rate of spontaneous abortion was significantly higher in the higher maternal age groups than in the lower groups.

In comparison to the data from the referenced study (Yan *et al.*, 2012), it was observed that among patients undergoing intracytoplasmic sperm injection (ICSI), there was only a trend of a negative association between maternal age and biochemical pregnancy. However, in patients undergoing *in vitro* fertilization (IVF), advanced maternal age did not exhibit an association with term birth, which is an unexpected finding. This discrepancy might be attributed to the small sample size of the study, potentially influencing the outcomes. Alternatively, it’s worth considering that 91.4% of the couples in the study had some form of diagnosed female infertility factor. Consequently, the impact of maternal age alone may not be as decisive in the results, particularly compared to a healthier population cohort. Further investigation and larger sample sizes may provide clarity on these observations.

## CONCLUSION

In conclusion, this study sheds light on the adverse impact of advanced paternal age on patients undergoing *in vitro* fertilization (IVF), particularly concerning the rate of full-term births. Its pioneering nature is noteworthy, given the limited availability of Brazilian data in this area. The findings underscore the significance of preconception public health advisories, emphasizing the risks associated with delaying parenthood for both men and women, especially among those in need of assisted reproductive techniques. By elucidating the diminished likelihood of full-term live births with advancing paternal age, such warnings can empower individuals to make informed decisions regarding family planning.

Moving forward, it is imperative to conduct further studies with larger sample sizes of IVF cycles to enable more robust analyses of advanced paternal age, independent of the strong influence of maternal age. By disentangling the effects of paternal age from maternal age, future research endeavors can provide deeper insights into the specific contributions of paternal age to fertility outcomes in assisted reproductive settings. These efforts are essential for refining clinical guidelines and informing personalized fertility treatment strategies tailored to the unique needs of patients.
